# Publisher Correction: Melting conditions in the modern Tibetan crust since the Miocene

**DOI:** 10.1038/s41467-018-06344-5

**Published:** 2018-09-17

**Authors:** Jinyu Chen, Fabrice Gaillard, Arnaud Villaros, Xiaosong Yang, Mickael Laumonier, Laurent Jolivet, Martyn Unsworth, Leїla Hashim, Bruno Scaillet, Guillaume Richard

**Affiliations:** 1 State Key Laboratory of Earthquake Dynamics, Institute of Geology, China Earthquake Administration, 100029 Beijing, China; 2 Université d’Orléans, CNRS, BRGM, ISTO, UMR 7327, F -45071 Orléans, France; 3 Laboratoire Magmas et Volcans, Campus Universitaire des Cézeaux, 6 Avenue Blaise Pascal, 63178 Aubière Cédex, France; 4 Sorbonne Université, CNRS-INSU, Institut des Sciences de la Terre Paris, ISTeP, UMR 7193, F-75005 Paris, France; 5 Department of Earth and Atmospheric Sciences, University of Alberta, Edmonton, AL T6G 2J1 Canada; 6 Department of Earth Science, University of Minnesota – Twin Cities, 55455 Minneapolis, MN USA

Correction to: *Nature Communications*; 10.1038/s41467-018-05934-7; published online 29 August 2018

The original PDF version of this Article contained an error in which Fig. 3 and its legend were omitted and Equations 5 and 6 contained errors.This has been corrected in the PDF version of the Article. The HTML version was correct from the time of publication.

Also in the original PDF version, there were errors in Equations 5 and 6. Both equations omitted all occurrences of *Φ*, and incorrectly read:

$$\left( {\upsilon _f - \upsilon _s} \right) = \frac{{k()}}{{\eta _f}}\delta \rho g$$

$$\lambda = \sqrt {\frac{{\eta _sk\left( {\,_0} \right)}}{{\eta _f0}}}$$

The correct forms of Equations 5 and 6 are:

$$\mathit{\Phi} \left( {\upsilon _f - \upsilon _s} \right) = \frac{{k\left( \mathit{\Phi} \right)}}{{\eta _f}}\delta \rho g$$

$$\lambda = \sqrt {\frac{{\eta _sk\left( {\mathit{\Phi} _0} \right)}}{{\eta _f\mathit{\Phi} _0}}}$$

This has been corrected in the PDF version of the Article. The HTML version was correct from the time of publication.

