# Supplementary material for: Melting conditions in the modern Tibetan crust since the Miocene
Source: Nat Commun. 2018 Aug 29;9:3515. doi: 10.1038/s41467-018-05934-7 (PMC6115434; doi:10.1038/s41467-018-05934-7)
Supplement: Supplementary file 1 — Supplementary Information [file 41467_2018_5934_MOESM1_ESM.pdf]

SUPPLEMENTARY INFORMATION

Melting conditions changes in the modern Tibetan crust since the Miocene

Jinyu Chen[1,2★], Fabrice Gaillard[2★], Arnaud Villaros[2], Xiaosong Yang[1], Mickael Laumonier[3], Laurent Jolivet[4], Martyn Unsworth[5], Leïla Hashim[6], Bruno Scaillet[2] & Guillaume Richard[2]

[1]State Key Laboratory of Earthquake Dynamics, Institute of Geology, China Earthquake Administration, Beijing 100029, China.

[2]Université d'Orléans, CNRS, BRGM, ISTO, UMR 7327, F -45071, France.

[3]Laboratoire Magmas et Volcans, Campus universitaire des Cézeaux, 6 avenue Blaise Pascal, 63178 Aubière Cédex, France.

[4]Sorbonne Université, CNRS-INSU, Institut des Sciences de la Terre Paris, ISTeP UMR 7193, F-75005 Paris, France

[5]Department of Earth and Atmospheric Sciences, University of Alberta, Edmonton, Alberta, T6G 2J1, Canada

[6]Department of Earth Science, University of Minnesota – Twin Cities, Minneapolis, Minnesota, USA

★email: jinyu@ies.ac.cn; fabrice.gaillard@cnrs-orleans.fr

Supplementary Note 1 | Mechanism for electrical transport

Assuming silicate melts are generally ionic conductors[1], a direct relationship between sodium mobility and electrical conductivity has been established by Gaillard[2], Ni *et al.*[3,4] and Laumonier *et al.*[5] and references therein. Na diffusivity (D_{Na}) was calculated from the Nernst-Einstein equation relating electrical conductivity and the tracer diffusion coefficient D_i (m^2/s) of an ion i:

$$D_i = \frac{\sigma_i \cdot q_i^2 \cdot N_i}{k \cdot T \cdot H_r} \quad (1)$$

wih σ_i being electrical conductivity (S/m) of the leucogranitic melts derived from P-T-H_2O conductivity model (equation (1)-(4) of the main text), q_i and N_i being respectively the charge (C) and the concentration (m^{-3}) of i, k is the Boltzmann constant (1.38×10^{-23} J/K), T is the temperature (K) and H_r is the Haven ratio. A value of 1.0 was taken for Hr following previous works on rhyolite[2,6] and albite[4]. $\log_{10} D_{Na}$ was plotted *vs.* 10,000/T in Supplementary Fig. 5. From previous studies, Na diffusivity of dry[7,8] or hydrous rhyolite melt[9] are close to the ones of dry leucogranitic melt at 1 atm and slightly lower than the hydrous melt calculated from equation (1). The diffusivity of potassium[8], being another important ionic species in the leucogranitic glass-melt, is about two orders of magnitude smaller than that of sodium (Supplementary Fig. 5). Therefore, we infer that sodium is the dominant charge carrier in the leucogranitic melt as stated in previous works[2,4-6,10,11].

Supplementary Note 2 | Comparison with previous work

The electrical conductivity of the leucogranitic melt calculated by equation (1)-(4) of the main text is plotted against the pressure (Supplementary Fig. 6a) and the water content (Supplementary Fig. 6b) at 1000 °C and compared with previous experimental works on silicate melts (*i.e.* metaluminous rhyolite[2,6], dacite[5] and andesite[12]). For hydrated magmas, increasing pressure reduces the possibility of interstitial cation migration in the melts and consequently decreases the electrical conductivity[2]. Because rhyolite and leucogranite melts are strongly polymerized[2,10], the effect of pressure is more important than for dacite[5] and andesite[12] (Supplementary Fig. 6a). The addition of water facilitates sodium mobility and then increases the electrical conductivity[2,10,11]. The effect of the water content on silica-rich melts is less important than for more mafic compositions. Our work compared with previous studies indicates that the effect of water is similar to that described by Gaillard[2] in the

range 0-3 wt% H_2O, while the extrapolation of the formalism of Gaillard[2] yield an underestimation of the effect of water at H_2O content >3 wt%. Finally, we see that electrical conductivity of rhyolitic melts do not significantly vary between peraluminous (*i.e.* Al > Na+K; this study), metaluminous (Gaillard[2]) and peralkaline (Al < Na+K; Guo *et al.*[6]) compositions.

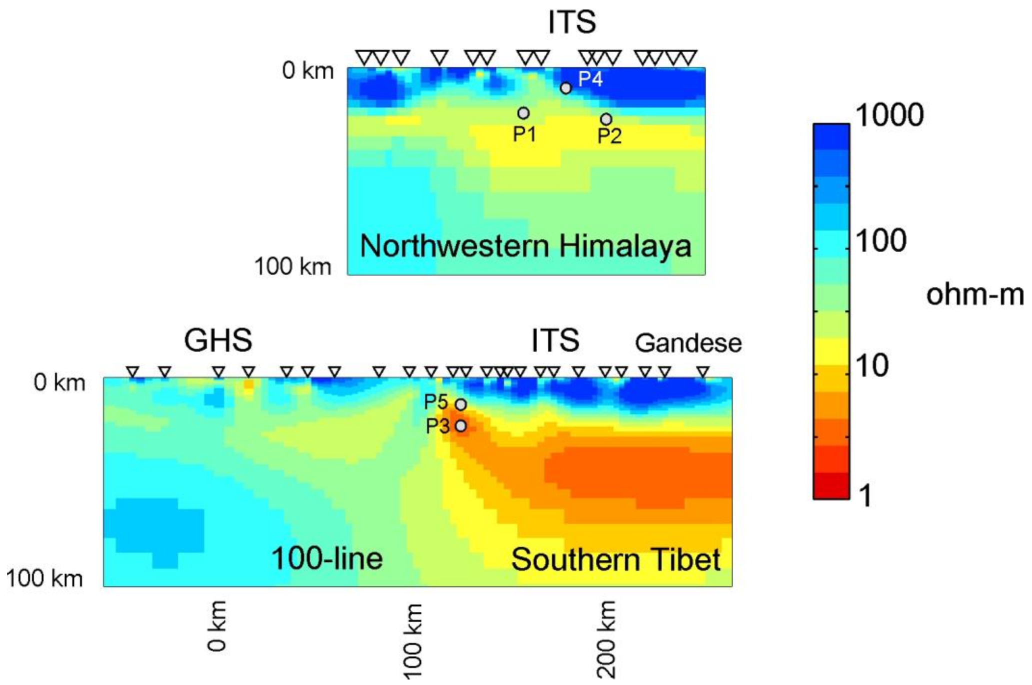

Supplementary Figure 1 | The 5 anomalously conductive points (P1-P5) labeled in Fig.1b-c positioned in the MT works of the northwestern Himalaya[13] and the southern Tibet[14].

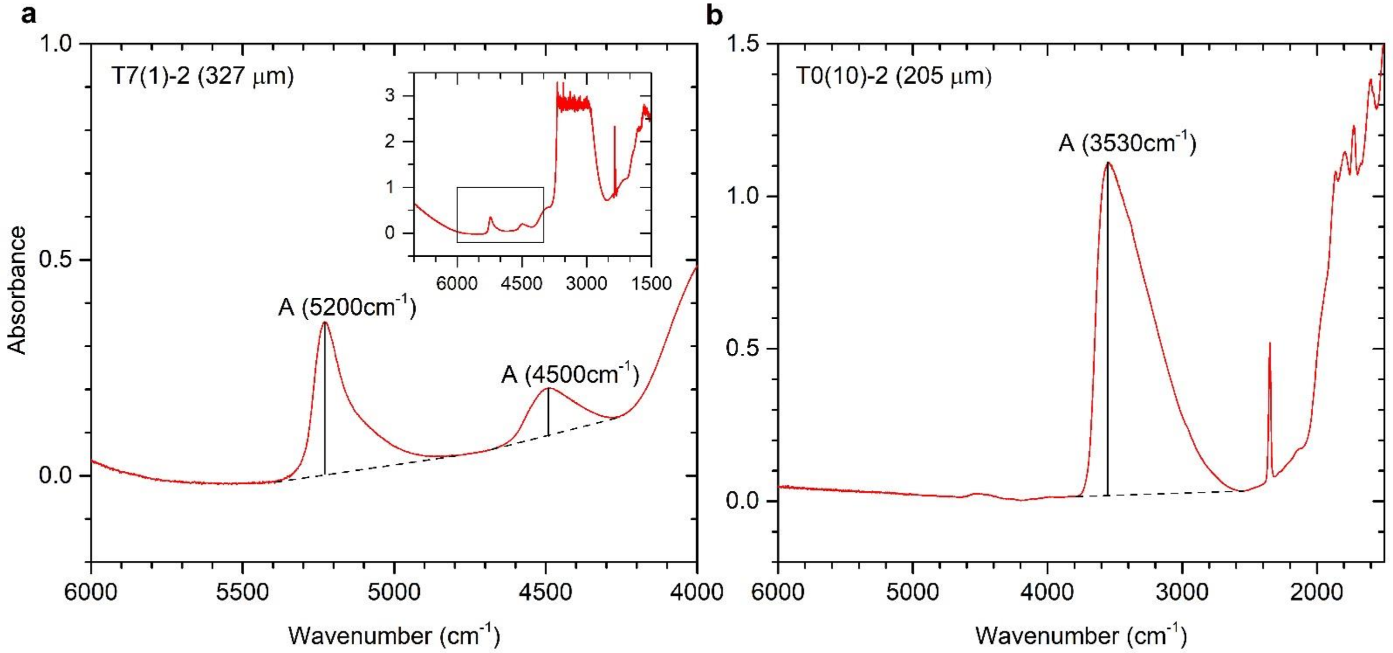

Supplementary Figure 2 | Typical FTIR spectra used to calculate the water concentration of samples after the experiments. a, sample containing 6.41 wt% H_2O [T7(1)-2]. **b**, sample containing 0.55 wt% H_2O [T0(10)-2].

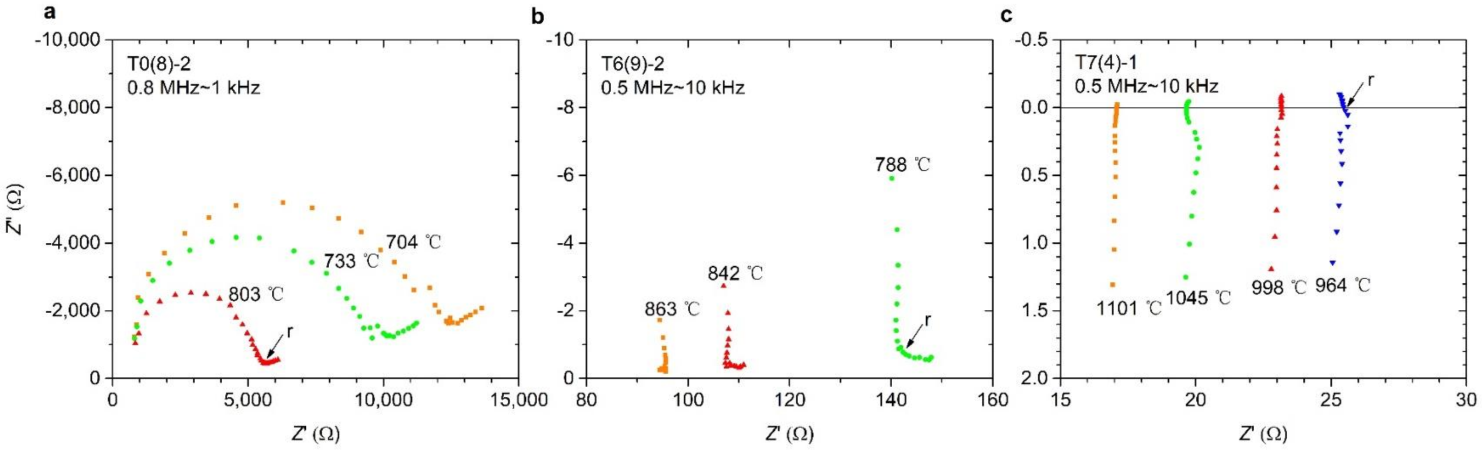

Supplementary Figure 3 | Nyquist plots of impedance spectra. The sample resistance (*r*) is obtained from the spectra intercept with the real axis (*Z*'). Resistance decreases as temperature increases. **a**, For *Z*'<r, the semicircles suggests relatively large resistance (>~10^3 Ω), *e.g.* dry samples at low temperature; for *Z*'>r, the linear part, indicates the effect of the interface between the sample and the electrode. **b**, Intermediate (~10^2 Ω to ~10^3 Ω) represents intermediate electrical response between (**a**) and (**c**). **c**, The nearly vertical line implies relatively small resistance (<~10^2 Ω), *e.g.* hydrous samples at high temperature.

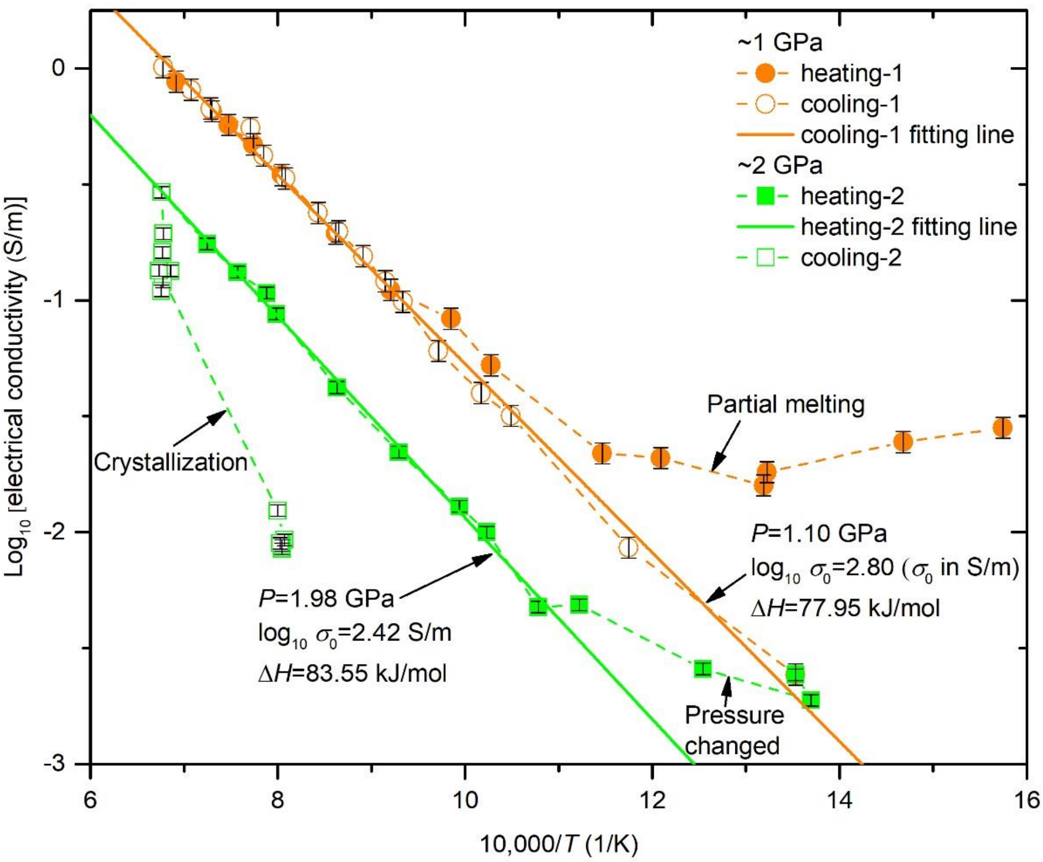

Supplementary Figure 4 | The electrical conductivity of sample T0(8) as a function of the reciprocal temperature at ~1 GPa and ~2 GPa (one heating-cooling cycle for each pressure). Dashed lines are the trajectory of the experimental process (initiating from room *T*), while straight full lines indicate the fit of experimental data at the two pressures investigated here (1.10 and 1.98 GPa). This figure illustrates the way data points were selected to proceed to the Arrhenius fit that are shown in Table 2. Note that crystallization occurred upon cooling at ~2 GPa, which was surprising given the well-known slow reactivity of such melts, but it explains well the electrical behavior.

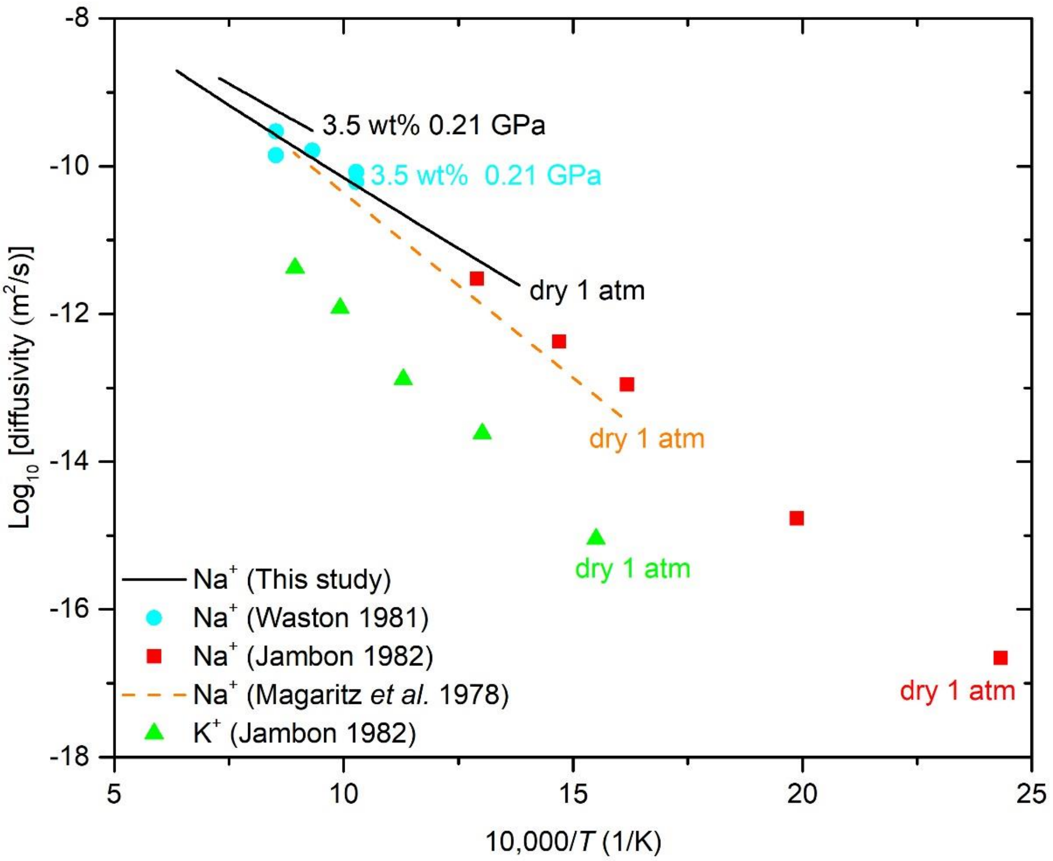

Supplementary Figure 5 | Na^+ diffusivities calculated from conductivity measured in this study (black solid lines) compared with diffusion measurements from other studies in felsic melts (color dashed lines or symbols). Na^+ diffusivity (black full line) was calculated by equation (1) in the temperature range of this study. The color dashed lines and symbols illustrate Na^+ diffusivities in dry melts at 1 atm[7,8], Na^+ diffusivities in melts containing 3.5 wt% water at 0.21 GPa[9] and K^+ diffusivities in dry melts at 1 atm[8].

a

b

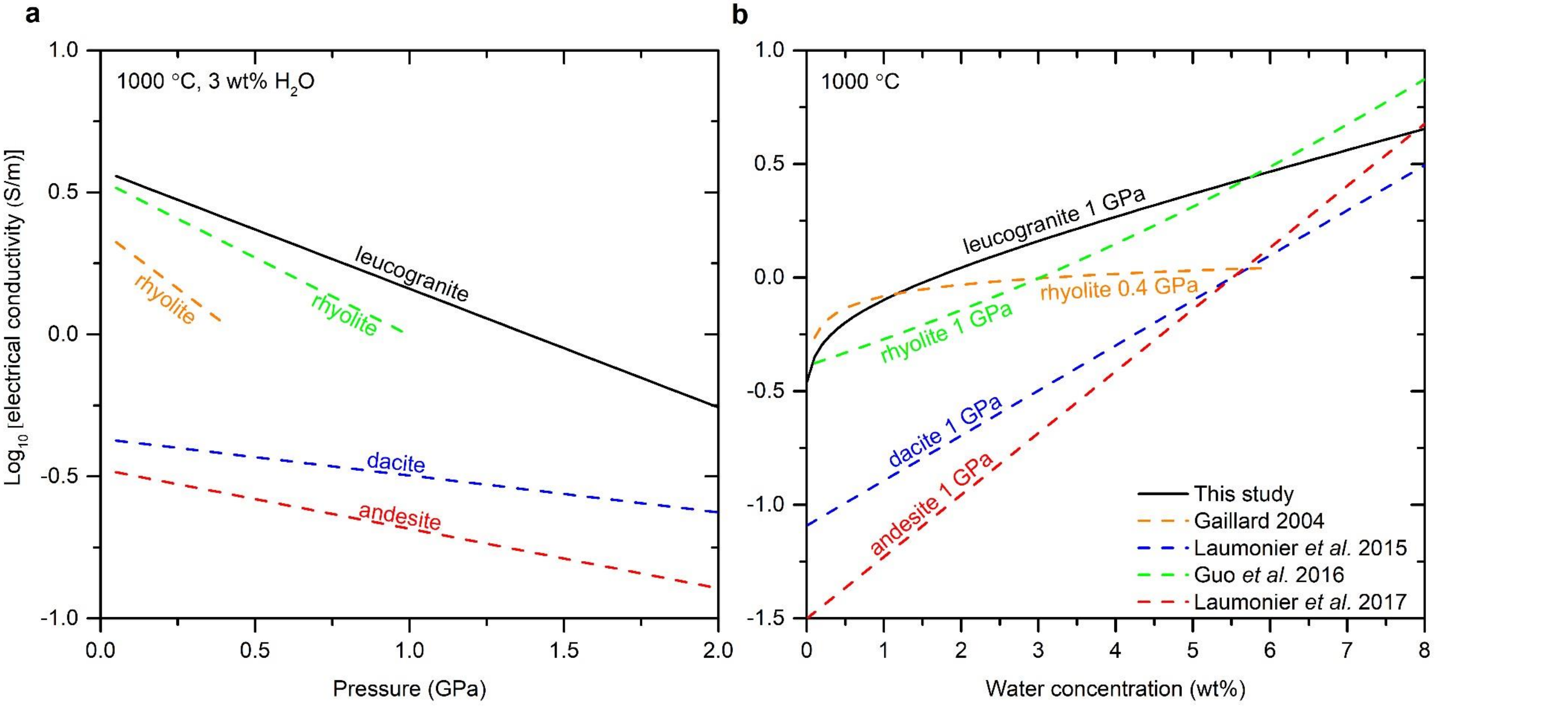

Supplementary Figure 6 | Log$_{10}$ of electrical conductivity *vs.* pressure (a) and water concentration (b) of the leucogranitic melts (straight lines) compared with other silicate melts (dashed lines). The other melts include rhyolite[2,6], dacite[5] and andesite[12].

Supplementary Table 1 | The depth of anomalous layers beneath the Himalayan orogeny gathered from the literature.

Location	Depth (km)	Characterisitic	Reference
Northwestern Himalaya	10~25	Low resistivity layer	Arora *et al*.[13]
Northwestern Himalaya	~30	Low velocity layer	Caldwell *et al*.[15]
Southern Tibet	<25	High heat flow	Francheteau *et al*.[16]
Southern Tibet	3~33	Low resistivity layer	Pham *et al*.[17]
Southern Tibet	10~20	Low resistivity layer	Chen *et al*.[18]
Southern Tibet	15~18	Seismic bright spots	Brown *et al*.[19]
Southern Tibet	~15	P-S wave conversion	Makovsky *et al*.[20,21]
Southern Tibet	15~20	Low resistivity layer	Wei *et al*.[22]
Southern Tibet	>10	Low resistivity layer	Li *et al*.[23]
Southern Tibet	10~30	Low resistivity layer	Unsworth *et al*.[14]

Supplementary Table 2 | The chemical compositions of the leucogranitic rocks (T12-22a and DK89a), the starting volatile-free glasses (T12-22b and DK89b) and the glasses after experiments. Chemical compositions (given in wt%) were determined by EMP, except T12-22a that was determined by XRF. Standard deviations are marked in parentheses below the compositions.

Samples	SiO_2	TiO_2	Al_2O_3	FeO	MgO	CaO	Na_2O	K_2O	P_2O_5	Total
					(1) Starting bulk rocks					
T12-22a	74.15	0.05	14.73	0.95[a]	0.17	0.74	4.46	3.95	0.13	99.40
DK89a[24]	73.04	0.13	15.32	0.91[a]	0.20	0.85	3.85	4.96	0.14	99.55
					(2) Dry starting glasses					
T12-22b	73.30	0.03	13.82	0.75	0.11	0.61	4.35	4.13	0.12	97.22
	(1.73)	(0.05)	(1.07)	(0.11)	(0.03)	(0.11)	(0.31)	(0.16)	(0.06)	(0.90)
DK89b[24]	73.87	0.17	15.50	0.77	0.19	0.84	3.75	4.91	nd	99.34
	(0.34)	(0.13)	(0.15)	(0.07)	(0.02)	(0.03)	(0.07)	(0.06)	nd	nd
					(3) Post-conductivity experiment glasses					
T0(10)-2	73.48	0.03	14.61	0.69	0.12	0.64	4.60	4.19	0.13	98.66
	(1.01)	(0.04)	(0.49)	(0.12)	(0.02)	(0.04)	(0.14)	(0.19)	(0.06)	(0.51)
T3(9)-2	71.08	0.03	13.99	0.75	0.11	0.63	4.15	4.12	0.10	95.08
	(1.62)	(0.04)	(0.56)	(0.18)	(0.03)	(0.07)	(0.44)	(0.27)	(0.06)	(1.20)
T6(9)-2	68.98	0.02	13.30	0.68	0.12	0.61	3.44	3.86	0.08	91.32
	(0.43)	(0.03)	(0.17)	(0.23)	(0.02)	(0.03)	(0.19)	(0.10)	(0.07)	(0.57)
T7(4)-1	68.25	0.03	13.02	0.68	0.11	0.59	2.86	3.78	0.08	89.59
	(0.49)	(0.04)	(0.23)	(0.19)	(0.02)	(0.04)	(0.15)	(0.10)	(0.07)	(0.72)

nd: not determined.

[a]FeO as total Fe ($FeO+Fe_2O_3$).

Supplementary Table 3 | Experimental details and results of the conductivity measurements. "Adjusted *P* (GPa)" and "Adjusted H_2O (wt%)" refer to calculated pressures and water concentrations obtained after the optimization described in the Methods (Modeling the electrical conductivity of melts).

Samples	*P* (GPa)	Adjusted *P* (GPa)	*T* (°C)	H_2O (wt%) range before experiments (points)	H_2O (wt%) range after experiments (points)	Adjusted H_2O (wt%)	Log_{10} σ_0 (S/m)	ΔH[a] (kJ/mol)
				(a) T12-22				
T0(11)-0.5	0.36-0.60	0.40	777-1200	nd	0.05-0.08(10)	0.07	2.7	64.5
T0(8)-1	0.90-1.37	1.10	466-1203	nd	nd	0.09	2.8	78.0
T0(8)-2	1.77-2.35	1.98	803-1206	nd	nd	0.09	2.4	83.5
T0(10)-2	1.81-2.47	2.03	1092-1305	nd	0.40-0.60(10)	0.55	2.5	79.8
T3(9)-2	1.86-2.46	2.22	798-1100	2.73-4.44(10)	1.26-4.63(8)	3.67	2.1	57.0
T6(9)-2	1.90-2.52	2.08	785-1105	5.01-7.50(10)	4.56-7.07(10)	5.92	2.6	56.1
T7(1)-2	1.90-2.47	2.24	760-1018	6.15-8.97(10)	5.45-8.10(7)	6.41	2.4	50.6
T7(4)-1	1.10-1.36	1.33	785-1101	6.29-9.12(10)	4.78-8.42(10)	7.90	2.3	41.5
				(b) DK89				
DK0a	0.94-1.14	1.14	745-1408	nd	nd	0.00	2.9	83.6
DK7a	0.98-1.20	1.19	909-1407	5.77-8.29(2)	0.93-2.61(5)	1.09	2.4	62.5
DK11b	0.96-1.18	1.17	791-1403	7.83-11.97(19)	1.05-1.71(9)	5.73	2.6	53.1

nd: not determined.

[a]The activation enthalpy (ΔH) includes the activation energy (E_a) and the activation volume (ΔV) as follows: $\Delta H = E_a + P \cdot \Delta V$.

Supplementary Table 4 | End members (C_0, *i.e.* fluid-absent, and C_1, H_2O-richest fluid-present) used to calculate the melting relationships, *i.e.* the melt percentage and melt water content as a function of *P* and *T*.

	SiO_2	TiO_2	Al_2O_3	FeO	MgO	CaO	Na_2O	K_2O	H_2O	Total
C_0	61.49	1.21	19.00	4.50	3.23	1.05	2.59	5.26	1.68	100.00
C_1	59.55	1.17	18.40	4.35	3.13	1.01	2.51	5.09	4.77	100.00

Supplementary References

1. Ni, H., Hui, H. & Steinle-Neumann, G. Transport properties of silicate melts. *Rev. Geophys.* **53,** 715-744 (2015).
2. Gaillard, F. Laboratory measurements of electrical conductivity of hydrous and dry silicic melts under pressure. *Earth Planet Sci. Lett.* **218,** 215-228 (2004).
3. Ni, H., Keppler, H. & Behrens, H. Electrical conductivity of hydrous basaltic melts: implications for partial melting in the upper mantle. *Contrib. Mineral Petrol.* **162,** 637-650 (2011).
4. Ni, H., Keppler, H., Manthilake, M. A. G. M. & Katsura, T. Electrical conductivity of dry and hydrous $NaAlSi_3O_8$ glasses and liquids at high pressures. *Contrib. Mineral Petrol.* **162,** 501-513 (2011).
5. Laumonier, M., Gaillard, F. & Sifre, D. The effect of pressure and water concentration on the electrical conductivity of dacitic melts: Implication for magnetotelluric imaging in subduction areas. *Chem. Geol.* **418,** 66-76 (2015).
6. Guo, X., Zhang, L., Behrens, H. & Ni, H. Probing the status of felsic magma reservoirs: Constraints from the *P*–*T*–H_2O dependences of electrical conductivity of rhyolitic melt. *Earth Planet Sci. Lett.* **433,** 54-62 (2016).
7. Magaritz, M. & Hofmann, A. W. Diffusion of Sr, Ba and Na in obsidian. *Geochim. Cosmochim. Ac.* **42,** 595-605 (1978).
8. Jambon, A. Experimental results for Na, K, Rb, Cs, Ca, Sr, Ba, Ce, Eu to 1300 °C and a model of calculation. *J. Geophys. Res.* **87,** 10797-10810 (1982).
9. Watson, E. B. Diffusion in magmas at depth in the earth: The effects of pressure and dissolved H_2O. *Earth Planet Sci. Lett.* **52,** 291-301 (1981).
10. Gaillard, F. & Marziano, G. I. Electrical conductivity of magma in the course of crystallization controlled by their residual liquid composition. *J. Geophys. Res.-Sol. Ea.* 110, B06204 (2005).
11. Pommier, A., Gaillard, F., Pichavant, M. & Scaillet, B. Laboratory measurements of electrical conductivities of hydrous and dry Mount Vesuvius melts under pressure. *J. Geophys Res.-Sol. Ea.* **113,** B05205 (2008).
12. Laumonier, M., Gaillard, F., Muir, D., Blundy, J. & Unsworth, M. Giant magmatic water

reservoirs at mid-crustal depth inferred from electrical conductivity and the growth of the continental crust. *Earth Planet Sci. Lett.* **457,** 173-180 (2017).

13. Arora, B. R., Unsworth, M. J. & Rawat, G. Deep resistivity structure of the northwest Indian Himalaya and its tectonic implications. *Geophys. Res. Lett.* **34,** L04307 (2007).
14. Unsworth, M. J. et al. Crustal rheology of the Himalaya and Southern Tibet inferred from magnetotelluric data. *Nature.* **438,** 78-81 (2005).
15. Caldwell, W. B., Klemperer, S. L., Rai, S. S. & Lawrence, J. F. Partial melt in the upper-middle crust of the northwest Himalaya revealed by Rayleigh wave dispersion. *Tectonophysics.* **477,** 58-65 (2009).
16. Francheteau, J. et al. High heat flow in southern Tibet. *Nature.* **307,** 32-36 (1984).
17. Pham, V. N. et al. Partial melting zones in the crust in southern Tibet from magnetotelluric results. *Nature.* **319,** 310-314 (1986).
18. Chen, L. et al. Electrically conductive crust in southern Tibet from INDEPTH magnetotelluric surveying. *Science*. **274,** 1694-1696 (1996).
19. Brown, L. D. et al. Bright Spots, Structure, and Magmatism in Southern Tibet from INDEPTH Seismic Reflection Profiling. *Science*. **274,** 1688-1690 (1996).
20. Makovsky, Y. et al. INDEPTH wide-angle reflection observation of P-wave-to-S-wave conversion from crustal bright spots in Tibet. *Science*. **274**, 1690-1691 (1996).
21. Makovsky, Y. & Klemperer, S. L. Measuring the seismic properties of Tibetan bright spots: Evidence for free aqueous fluids in the Tibetan middle crust. *J. Geophys. Res.-Sol. Ea.* **104,** 10795-10825 (1999).
22. Wei, W. et al. Detection of widespread fluids in the Tibetan crust by magnetotelluric studies. *Science.* **292,** 716-719 (2001).
23. Li, S. et al. Partial melt or aqueous fluid in the mid-crust of Southern Tibet? Constraints from INDEPTH magnetotelluric data. *Geophys. J. Int.* **153,** 289-304 (2003).
24. Scaillet, B., Pichavant, M. & Roux, J. Experimental Crystallization of Leucogranite Magmas. *J. Petrol.* **36,** 663-705 (1995).
